# Supplementary material for: Plasmodium falciparum protein phosphatase PP7 is required for early ring-stage development
Source: mBio. 2024 Oct 10;15(11):e02539-24. doi: 10.1128/mbio.02539-24 (PMC11559042; doi:10.1128/mbio.02539-24)
Supplement: Supplemental material — Supplemental figures; captions for supplemental tables and movie. [file mbio.02539-24-s0001.docx]

**Supplementary Figure S1**

(A)

MENYNIEDVVMIYGHKAIIESIQNVENVKSEIQPNKIYVGKYVNKKGYSDGTYRKKKIYTPQNDELVILCSYNSIKPY**NE**

**EESACTMIQKMFRGYQGRKSFH**SFVCCTVWRKFDHIHEYITLNNHDEIYKPLIKTIKRDIKKGIIQFPSKCFSSNSNQFS

RVSTTSYESSNYNENV**PRLKDKIDRTFATEMFHFFLTSKEIILPYNLVHKVLIKTKKMLEENIKSSVINLDMSKKSKDTK**

**LIILGDVHGQLHDVLWLFNRFGIPSSTNIYIFNGDIADRGENATEIFILLFIFKLSCNDSVIINRGNHECSYMNEVYGFY**

**NEVLSKYDNTIFDLFQNIFELLGLAVNVQNQIFVVHGGLSRYQDITLKEIDELDRKKQEILHPEQYEDIVIFDLLWSDPQ**

**KKEGIGGNARGNNCITFGPDVTEMFLKNNNLDILIRSHQVPKTLKGIESHHEGKCITLFSASNYCNKIKNLGAAIIFNQD**

LTFEVQEYMSPSLEVIRETFEENQKLREKVLHCSKIVELEKNEQKNNNKLSTEGLMNDIINCLSTIICNEKNSLWNNLYK

QDKDKKGVVHINIWKEELGKLSKAKKVPWIYLCRKLKMIEDYHVNYNNILSRFKINYAPNEKFLNTEWKNECFEHLYEAL

LKADLS**LRETLMVFDKNLDGKVSFAEFEQVLRDLNIDLSNEQIRILVRLINSNSLCNNTNLQENDKIDVAEFI**GKMRVCY

RLSINKDYVNNEKIQKLIETIGKHILSDSADTANYHYKFYEENNERHNSERRKRSSVIKSV**ALFQKFKNYDNFGNGYLDY**

**NDFVKAIKNFDMNKISKEVEFEVDDDILMELAKSIDITKSSKINFLEFLQAFY**VVNKSKYSYVDEIWCHICTVIYENKVA

LKKCMKYLEDTMQGKITSIHFRYILLELNKILQEHNFEMNKPLTDEQIDLLAYTVETDDKVDYVEFFNSFKPTYIYSNN

(B)

PfPP7 IKPYNEEES**A**CTM**IQ**KMF**R**G**Y**QG**R**KSFHSFVCCTVWRKFDHI 116
PvPP7 IRPFSETES**A**SIR**IQ**RAF**R**G**Y**R**AR**KEFHSFVSSLVWRKFEHL 116

TgPP7 ARPYDEKDCSATQL**Q**AAW**R**S**Y**R**AR**QAFQDAAAFNFWNELDAF 118

DmrdgC PPDLETTIR**A**AIF**IQ**KWY**R**RHQ**AR**REMQRRCNWQIFQNLEYA 56
HsPPEF1 TRRSDTSLR**A**ALI**IQ**NWY**R**G**Y**K**AR**LKARQHYALTIFQSIEYA 51

. :. :* :* ::.* : .:..::

(C)

PfPP7 SKCFS**S**NSNQFSRV----STTSYESSNYNENV**P**RLKDK**I**DRTFATEMFHF**F**LTSKEII**L**P 204
PvPP7 SKCFS**S**NSNQFSRI----STTSYESTAYDENV**P**KLKDK**I**DKAFAMEVFHF**F**LTTKNYV**L**P 204

TgPP7 SRRRS**S**TRRSFGRSSVDASSISAENIPDDYAG**P**RVKWP**I**TREFCLDLVDHYRDNPDVP**L**P 238

DmrdgC GSAHV**S**VLDDKDDL----VE-----EFGDIVNAKIELP**I**RKNHIDLLIDV**F**RKKRGNR**L**H 137
HsPPEF1 NQSLE**S**EQDMRDRW----DYVDSIDVPDSYNG**P**RLQFPLTCTDIDLLLEA**F**KEQQ--I**L**H 135
 . * . . ::: : :.. : *

**D** **HGQ** **D** **G**

PfPP7 YNL**V**HKV**L**IKTK**K**M**L**EENIKSSVINLDMS**K**KSKDTKLIIL**GD**V**HG**Q**L**H**D**V**L**WL**F**NRF**G**I**P** 264
PvPP7 NNL**V**CKI**L**TKTQ**K**M**L**EENIKSSVIHVDMT**K**KSKDTKLIIL**GD**V**HG**Q**L**N**D**V**L**WL**F**NRF**G**L**P** 264

TgPP7 RKYALELIMAIADHYRETMKGAVVEVEIP**K**KG-GSRLVLV**GD**T**HG**Q**L**N**D**V**L**WI**F**YKF**G**P**P** 297

DmrdgC PKY**V**ALI**L**REAA**K**S**L**KQLPNISPVSTAVSQQ-----VTVC**GD**L**HG**K**L**D**D**L**L**VVLHKN**G**L**P** 192
HsPPEF1 AHY**V**LEV**L**FETK**K**V**L**KQMPNFTHIQTSPS**K**E-----VTIC**GD**L**HG**K**L**D**D**LFLI**F**YKN**G**L**P** 190

: . :: . .: : : : :: : : ** **:*.*:: :: : * *

**F** **GD** V**DRG** S  **K**  **RGNHE F E**
PfPP7 **S**ST**N**I**Y**I**FNGD**IA**DRG**ENAT**EI**FIL**L**FIFK**L**SCNDS**V**II**NRGNHE**CSY**MN**EV**YGF**YN**E**VL 324

PvPP7 **S**SN**N**I**Y**I**FNGD**IA**DRG**ENAT**EI**FLL**L**FTFK**L**SNYDC**V**II**NRGNHE**CSY**MN**EV**YGF**YN**E**VL 324

TgPP7 **S**AT**N**V**Y**L**FNGD**IA**DRG**RYAV**EI**FMM**L**FAFK**L**QCPSS**V**VI**NRGNHE**SAD**MN**EV**YGF**AQ**E**VR 357

DmrdgC **S**SS**N**P**Y**V**FNGD**FV**DRG**KRGL**E**VLLL**L**LSLY**L**AFPNA**V**FL**NRGNHE**DSV**MN**AR**YGF**IR**E**VE 252
HsPPEF1 **S**ER**N**P**Y**V**FNGD**FV**DRG**KNSI**EI**LMI**L**CVSF**L**VYPNDLHL**NRGNHE**DFM**MN**LR**YGF**TK**E**IL 250

* * *:****:.***. . *::::* * . : :****** ** *** .*:

**F** **HGG**   **R** P

PfPP7 S**KY**---DNTIFDLFQNIFEL**L**G**L**AVNVQNQIF**VVHGG**LSRYQDIT**L**KE**I**DELD**R**K**K**QEI- 380

*PvPP7*  S**KY**---DSAVFDLFQGVFEL**L**G**L**AVNIQNQIF**VVHGG**LSRYQDLTMRE**I**DELD**R**K**K**HEI- 380

*TgPP7*  Q**KY**---GGFMYQKFQEVFHL**L**P**L**CVVMEKRVF**VVHGG**LCRKDNVT**L**QH**I**DRLN**R**QRPCP- 413

DmrdgC S**KY**PRNHKRILAFIDEVYRW**L**P**L**GSVLNSRVLI**VHGG**FSD--STS**L**DL**I**KSID**R**G**K**YVSI 310
*HsPPEF1* H**KY**KLHGKRILQILEEFYAW**L**PIGTIVDNEIL**V**I**HGG**ISE--TTD**L**NLLHRVE**R**N**K**MKSV 308

** : :: .: * : ::..::::***:. : :. ::* :

G **W D**

PfPP7 -------------------------------------LHPEQY**E**DIV**IFD**L**LWSDPQ**KKE 403
PvPP7 -------------------------------------LHPEKY**E**DTI**IFD**L**LWSDPQ**KKN 403

TgPP7 -------------------------------------ACPHSF**E**DTLM**FD**L**LWSDPQ**HES 436

DmrdgC LRPPLTD--------------------------G---EPLDKT**E**WQQ**IFD**IM**WSDPQ**ATM 341
HsPPEF1 LIPPTETNRDHDTDSKHNKVGVTFNAHGRIKTNGSPTEHLTEH**E**WEQ**I**I**D**I**LWSDP**RGKN 368

. * ::*::****:

**RG** F **R H** **G** **T**  **SA**P
PfPP7 **G**IGG**N**A-**RG**NN**C**IT**FGPD**V**T**EM**FL**KNNN**L**DIL**IRSH**QVPKTLK**G**I**E**SH**H**E**GK**CI**T**L**FSAS** 463
*PvPP7*  **G**IGG**N**A-**RG**NN**C**IT**FGPD**I**T**DL**FL**KKNNFDIL**IRSH**QVPKTLK**G**I**E**SH**H**E**GK**CI**T**L**FSAS** 462

*TgPP7*  **G**RGWST-**RG**AD**C**IA**FGPD**I**T**DA**FL**TKNN**L**EVC**IRSH**QVPTNLR**G**F**E**PV**H**D**G**RCV**T**L**FSAS** 495

DmrdgC **G**CVP**N**TL**RG**AGVW-**FGPD**V**T**DN**FL**QRHR**L**SYV**IRSH**ECKP--N**G**H**E**FM**H**DN**K**II**T**I**FSAS** 398
HsPPEF1 **G**CFP**N**TC**RG**GG**C**Y-**FGPD**V**T**SKI**L**NKYQ**L**KML**IRSH**ECKP--E**G**Y**E**IC**H**D**GK**VV**T**I**FSAS** 425
 * .: ** ****:*. :* . .:. ****: .* * *:.: :*:****

**NY N**
PfPP7 **NY**CNKIK**N**L**GA**A**I**IFNQD**L**TFEVQE**Y**MS 490
*PfPP7*  **NY**CNKIK**N**L**GA**A**I**VFNQD**L**TFEVQE**Y**MS 490

TgPP7 **NY**CGTTG**N**F**G**GV**I**IFEAN**L**SFEIQE**Y**MA 525

DmrdgC **NY**YAIGS**N**K**GA**Y**I**RLNNQ**L**MPHFVQ**Y**IS 426
*HsPPEF1* **NY**YEEGS**N**R**GA**Y**I**KLCSGTTPRFFQ**Y**QV 453

** * *. * : .. :*

(D)

PfPP7 ADLSLRETLMVFDKNLDGKVSFAEFEQVLRDLNIDLSNEQIRILVRLINSNSLCNNTNLQ 702
PvPP7 ADLSLRETLMVFDKNLDGKVSFAEFEQVLKDLNIDLSNEQIRILVRLINSNSLCNQDSSQ 702

TgPP7 ADLSLKETLMLFDRNCDGTVSFREFNELITELDVGLSEPQVRILMRLITASPAFNA---- 729

DmrdgC ------------------------------------------------------------ 529
*HsPPEF1* ------------------------------------------------------------ 555

PfPP7 ENDKID**V**A**E**FIGKMRVCYRLSINKDYVNNEKIQKLIETIGKHILSDSADTANYHYK-FYE 761
PvPP7 ESDKID**V**A**E**FIGKMRVCYRLAINKEYTNNEKIRKLIETIGKHILADSADTANFHYR-FYE 761

TgPP7 AAGSID**V**A**E**FLGRFRVVYSHVINDDKRSVPWLQRALHCIGKAILADKAEAANRHYEQQRQ 789

DmrdgC EADGMS**V**MDALYAN---------------------------------------------- 543
HsPPEF1 AH--STLV**E**TLYRY---------------------------------------------- 567

: : :

PfPP7 ENNERHNSERRKRSSVIKSVA**L**FQK**F**KNY**D**NFGN**G**YLDYND**F**VKA**IK**NFDMNKISKEVEF 821
PvPP7 ENNESAGSERRKRSSVIKSVA**L**FQK**F**KNY**D**NFGN**G**YLDYDD**F**VRA**IK**NFDMNKISKEVEF 821

TgPP7 DGNLGPDLNTRRRSSAVRAVA**L**FQK**F**KDYNEGGD**G**YLSYAD**F**VTG**IK**RLTIDE--EELGF 847

DmrdgC ------------------KAS**L**VAI**F**NII**D**ADNS**G**EITLDE**F**ETA**I**DLLVAHM-----PG 580
HsPPEF1 ------------------RSD**L**EII**F**NAI**D**TDHS**G**LISVEE**F**RAMW**K**LFSSHY-----NV 604

* *: : .* : :* . : .

PfPP7 EVD**D**DILME**LA**KSI**D**ITKSSK**I**NFL**EFL**Q**AF**Y**VV**N-KSKYSYVDEIWCHICTVIYEN**K**VA 880
PvPP7 DVD**D**EILME**LA**KSI**D**ITKSSK**I**NFL**EFL**Q**AF**Y**VV**N-KSKYSYVDEIWCHICTVIYEN**K**VA 880

TgPP7 ALT**D**EHLYK**VA**EAV**D**TTGSKR**I**NYL**EFL**Q**AF**H**VV**DSNSNNSAAEELWGQICTAIFQH**K**SS 907

DmrdgC AYSKAEMLEKCRMM**D**LNGDGKVDLN**EFL**E**AF**RLSDLHRKEQQDENIRRRSTGRPSVA**K**TA 640
HsPPEF1 HID**D**SQVNK**LA**NIM**D**LNKDGS**I**DFN**EFL**K**AF**Y**VV**HRYE-----------DLMKPDVTNLG 653
 . : : .. :* . . :: ***:** : . : .

**Domain architecture and similarity of *P. falciparum* PP7 to EF-hand-containing protein phosphatases of higher eukaryotes**

Although the name PP7 was coincidentally attributed to phosphatases from both animals and plants, it has been suggested that they are not orthologues and that the PP7 nomenclature is best used for the plant phosphatases of this PPP sub-family and PPEF for the animal phosphatases(Andreeva and Kutuzov, 2009). PPEFs also have an IQ-type calmodulin-binding domain upstream of the catalytic domain. Plant PP7s have a different calmodulin-binding domain that forms an insert within the catalytic domain (Kerk et al., 2021).

(Supp 1A) Amino acid sequence of the *P. falciparum* PP7 (PF3D7_1423300) showing its conserved domains. The IQ calmodulin-binding domain is shown in red, the phosphoprotein phosphatase (PPP) catalytic domain in blue and the EF-hand domains in green. These coloured sequences are boxed in panels B-D.

(Supp 1B) A Clustal alignment of the IQ (calmodulin-binding) domain of *P. falciparum* PP7 with the *P. vivax* and *T. gondii* orthologues, and also with two protein phosphatases of higher eukaryotes which have the same domain architecture: the *Drosophila melanogaster* retinal degradation serine/threonine phosphatase rdgC and its human orthologue PPEF1. Residues shown in bold red are identical in all five sequences, those in bold black are identical in four of the five sequences. The Clustal assignment of identical and conserved residues between the five sequences are shown below the alignment.

(Supp 1C) A Clustal alignment, with minor manual modification, of the same five proteins showing the protein phosphatase catalytic domain. Residues shown in bold blue are identical in all five sequences, those in bold black are identical in four of the five sequences. Of the 44 residues conserved across PPP families, as defined previously (Barton et al., 1994), 38 are present in PfPP7 and are shown in bold red above the alignment, and the six not conserved are shown in black. Motifs that are highly conserved amongst all eukaryotic PPP motifs, as previously depicted, (Kerk et al., 2021) are boxed with the positions of metal-binding residues highlighted in green and those involved in catalysis and arginine residues that interact with the substrate, highlighted in yellow). The Clustal assignment of identical and conserved residues between the five sequences are shown below the alignment. The phosphatase catalytic domain of PfPP7 is boxed and corresponds to the sequence coloured blue in panel A.

(Supp 1D) A Clustal alignment of the same five proteins showing the EF-hand domains. Residues shown in bold green are identical in all five sequences, those in bold black are identical in four of the five sequences. The Clustal assignment of identical and conserved residues between the five sequences are shown below the alignment.

**Supplemental Figure 2:**

**
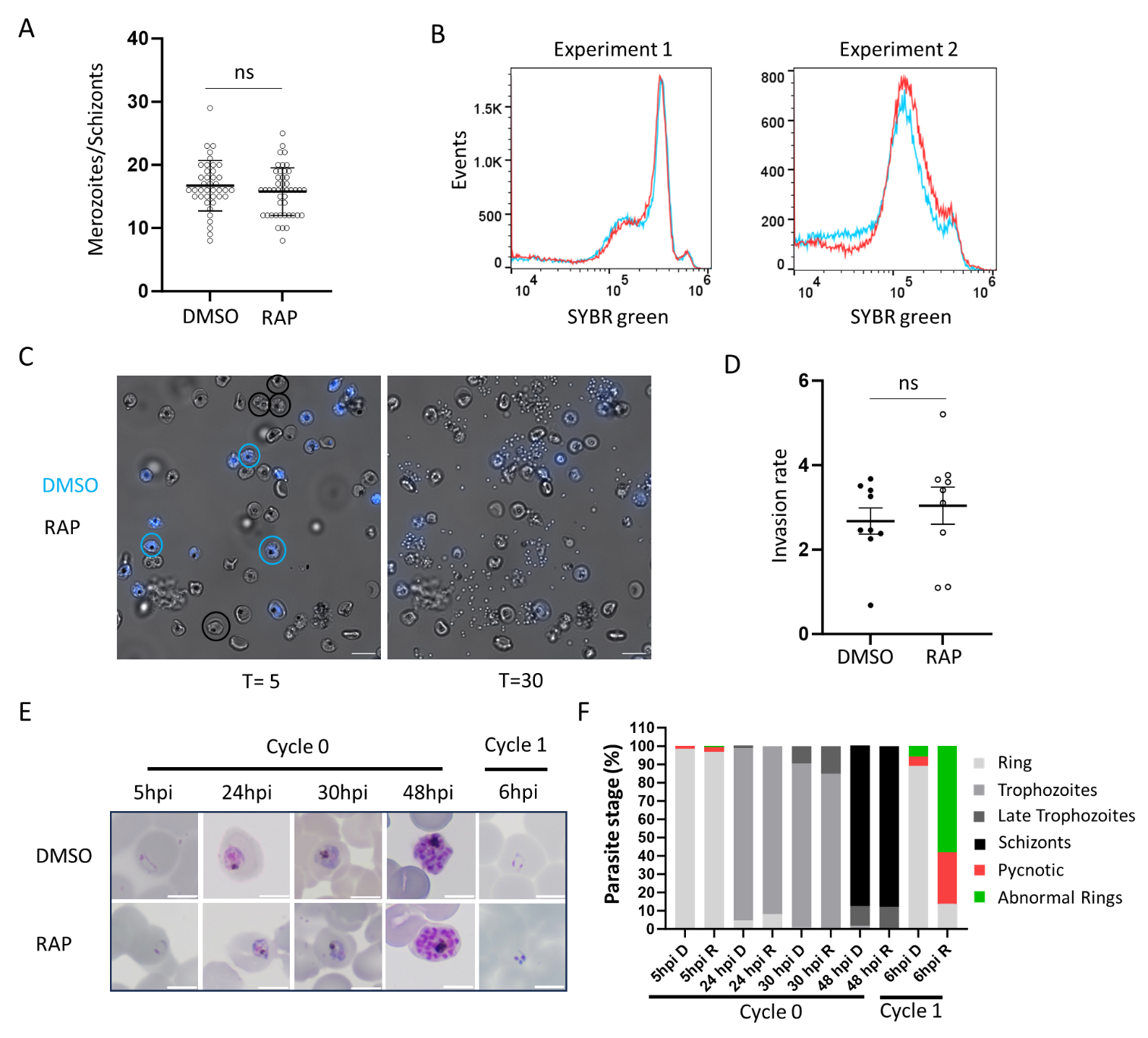
**

(Supp 2A) Number of merozoites per schizont in PP7-HA:loxP parasites treated with DMSO or rapamycin. Results are the average number of merozoites per schizont of two biological repeats (a minimum of 15 schizonts were counted per replicate). Error bars, SD. Mann–Whitney U test was performed for statistical analysis (ns: not significant).

(Supp 2B) DNA content of DMSO and rapamycin-treated PP7-HA:loxP schizonts. The histograms are representative of two independent experiment. At least 100,000 events were counted per sample. No obvious difference in the DNA content was detected.

(Supp 2C) Combined DIC and fluorescence images from time-lapse video microscopy of DMSO- and RAP-treated PP7-HA:loxP schizonts taken at 5 min (T = 5) and 30 min (T = 30) after release from compound 2. DMSO parasites were pre-treated with Hoechst to stain the nuclei so that they could be viewed simultaneously with the RAP-treated parasites in the same imaging chamber. Schizonts visible in the first frame that rupture over the course of the video are circled in blue (DMSO) or white (RAPA). Scale bar, 10 μm.

(Supp 2D) Invasion rate of DMSO and rapamycin-treated PP7-HA:loxP parasites. The parasitaemia of schizonts incubated with fresh erythrocytes was determined immediately (0 h) and after 4 hours post re-invasion. The invasion rate was determined as the ratio of the parasitaemia at the two time points (4 h/0 h). Three biological replicates were performed in triplicate. Error bars, error of mean. Mann–Whitney U test was performed for statistical analysis (ns: not significant).

**Supplemental Figure 3:
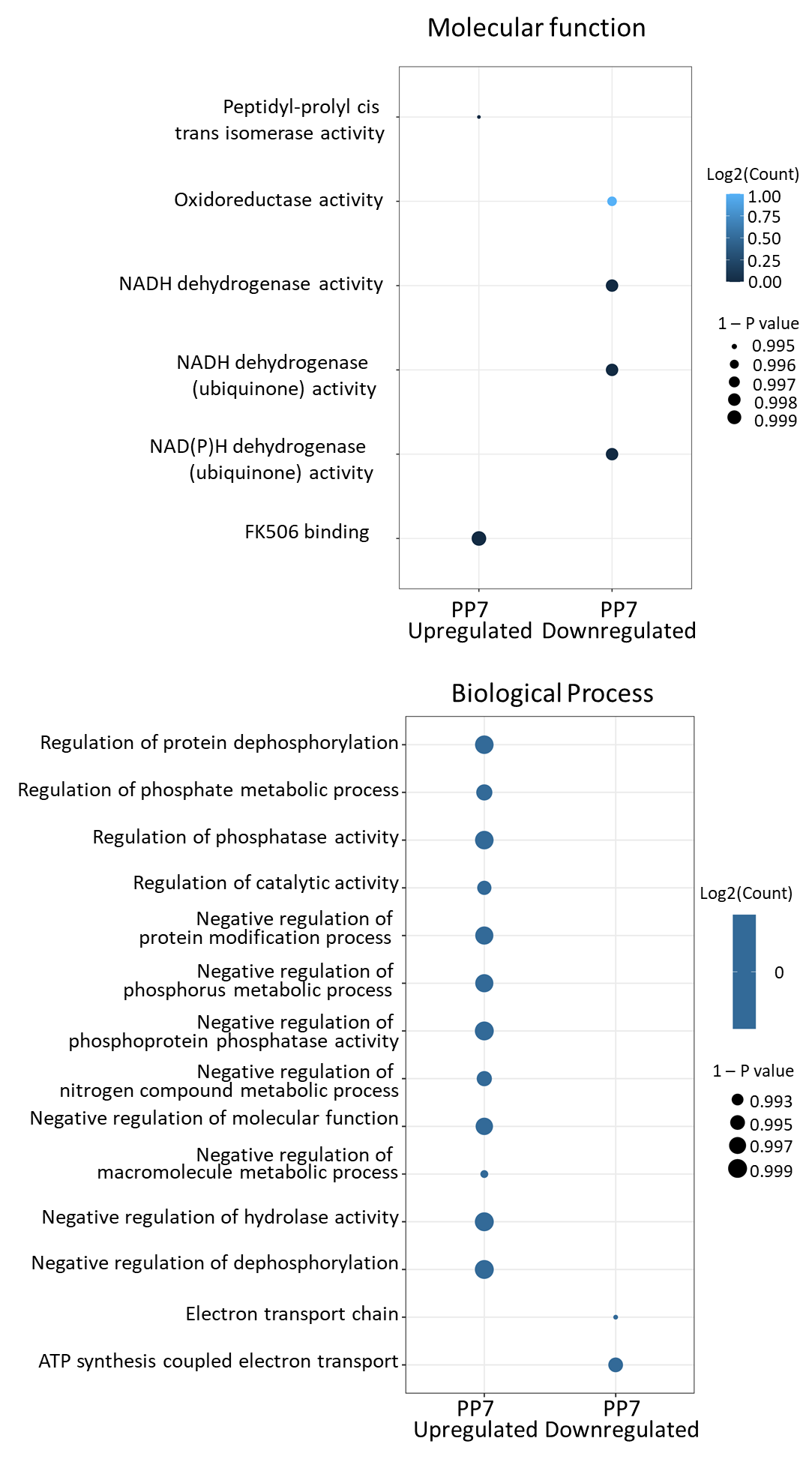
**

Gene ontology analysis performed on the DMSO and rapamycin-treated PP7-HA:loxP schizonts phosphoproteomes. Gene ontology (GO) enrichment analysis of significantly hyper or hypo phosphorylated proteins in DMSO and rapamycin-treated PP7-HA:loxP schizonts. GO terms were sourced from the PlasmoDB database. The level of significance (1-p value) of the enriched GO term is indicated by the size of the bubble. The colour density indicates the number of differentially expressed proteins (log2 protein count) associated with the GO term.

**Supplementary Figure S4**

(A)

ATATTAGGAGACGTTCATGGACAGTTACATGATGTCTTGTGGCTGTTTAACAGGTTCGGAATTCCGTCCTCAACCAACATCTACATCTTCAACGGTGACATCGCGGATAGGGGTGAAAACGCGACAGAGATCTTTATCCTATTGTTCATCTTTAAACTTAGCTGTAACGACTCTGTGATAATAAACAGAGGCAATCATGAGTGTAGTTACATGAACGAGGTTTACGGCTTCTACAATGAGGTTCTTTCTAAGTATGATAATACGATCTTTGACCTATTTCAAAATATTTTTGAACTACTGGGATTAGCAGTGAATGTACAGAACCAAATATTCGTGGTTCATGGTGGCCTTTCAAGATACCAGGACATAACCCTGAAGGAAATCGACGAGTTAGATAGAAAAAAACAGGAAATTCTTCACCCGGAGCAGTATGAAGACATAGTTATCTTCGATCTGTTGTGGTCTGACCCCCAGAAGAAAGAAGGCATCGGCGGTAATGCGCGTGGGAATAATTGTATAACCTTTGGGCCAGATGTCACAGAAATGTTCTTGAAAAATAATAATCTGGACATTCTTATTAGGTCACATCAGGTGCCAAAGACGCTAAAAGGCATAGAATCCCACCACGAAGGAAAATGTATAACGCTGTTCAGCGCCAGTAATTATTGTAACAAGATAAAAAACTTAGGGGCTGCTATAATATTTAATCAAGATCTTACTTTCGAGGTACAGGAATATATGTCCCCCTCCTTAGAAGTCATTAGGGAAACATTTGAGGAGAACCAGAAACTTAGAGAAAAGGTGTTGCACTGCTCCAAAATAGTGGAACTTGAAAAGAACGAACAGAAAAACAACAACAAGTTGAGCACTGAGGGTCTTATGAATGATATAATCAACTGTTTGTCAACAATCATCTGCAACGAGAAAAATAGTTTATGGAACAATTTGTATAAACAAGATAAAGACAAGAAAGGTGTAGTACATATAAATATCTGGAAGGAAGAGTTGGGTAAACTGAGTAAAGCAAAGAAGGTCCCTTGGATTTACTTGTGTAGAAAGTTAAAAATGATAGAAGACTATCATGTAAATTACAATAATATACTAAGCAGATTCAAAATAAACTATGCGCCAAATGAGAAGTTTCTAAACACAGAATGGAAAAATGAATGTTTCGAGCATCTATATGAGGCTCTTCTAAAAGCCGACTTATCCTTGAGAGAGACTTTAATGGTTTTTGACAAGAATCTGGACGGCAAGGTAAGCTTTGCAGAATTTGAACAGGTTTTAAGGGACCTAAACATAGATTTATCAAATGAACAAATCCGTATTCTTGTCAGGCTAATAAATTCCAACTCCTTATGCAATAATACAAACTTGCAGGAAAACGACAAGATCGACGTAGCGGAGTTTATAGGCAAAATGAGGGTTTGTTACAGACTTAGTATTAACAAGGACTATGTTAACAATGAGAAGATTCAAAAACTGATAGAAACAATCGGCAAACATATATTAAGCGATTCAGCTGATACTGCTAACTACCATTATAAATTCTACGAGGAGAACAACGAGAGACATAATTCCGAGAGAAGAAAGCGTAGCTCCGTAATTAAGTCCGTGGCCCTTTTTCAGAAATTCAAAAACTACGATAACTTCGGTAACGGTTATTTAGACTATAATGACTTCGTCAAGGCGATAAAAAACTTTGACATGAATAAAATTTCAAAAGAAGTCGAGTTCGAGGTAGACGACGATATCCTTATGGAGTTAGCAAAGTCCATAGATATCACAAAATCCAGTAAAATAAATTTTTTGGAGTTTTTGCAAGCTTTCTACGTGGTAAATAAGTCTAAGTACTCATACGTGGACGAGATTTGGTGCCACATCTGCACCGTGATCTATGAAAATAAGGTCGCCCTTAAAAAGTGTATGAAGTACCTTGAGGACACAATGCAGGGGAAAATTACAAGTATTCATTTTCGTTACATTCTTCTAGAGTTGAACAAAATCCTACAGGAGCACAACTTCGAGATGAATAAGCCGCTGACTGACGAGCAAATTGATTTGCTGGCGTATACAGTTGAGACGGACGATAAAGTGGATTATGTAGAGTTCTTCAACAGCTTCAAACCTACATATATATACAGCAATAAT

A ~800-bp 5′ homology region comprising the native gene sequence upstream of the re-codonised region was cloned upstream of the SERA2loxPint. Following transfection of purified schizonts using an AMAXA nucleofector 4D (Lonza) and P3 reagent (Lonza), modified parasites were selected as described previously (Birnbaum et al., 2017).

(B)

TTTTCTTCCCACATTTCGAATAAAACGCGTATGGTGTCCAAGGGCGAGGAAGACAACATGGCATCTTTGCCTGCCACTCACGAGTTGCATATTTTTGGATCAATTAACGGAGTGGATTTCGATATGGTTGGACAAGGGACGGGCAATCCAAATGATGGCTATGAAGAATTAAACTTGAAAAGCACGAAAGGTGATCTTCAGTTCTCCCCATGGATACTTGTCCCACATATTGGCTATGGGTTTCATCAATACTTGCCGTACCCGGATGGGATGTCCCCGTTTCAGGCAGCTATGGTCGACGGTAGCGGTTATCAGGTGCACAGAACAATGCAATTCGAGGATGGGGCCTCCTTGACGGTCAATTACAGGTACACGTATGAGGGAAGTCACATTAAAGGAGAGGCCCAAGTTAAAGGAACAGGTTTTCCTGCCGATGGACCGGTAATGACGAACAGCCTGACTGCCGCAGATTGGTGTAGGTCTAAAAAAACATACCCTAATGACAAGACAATAATTAGCACCTTTAAGTGGAGCTATACAACTGGTAATGGGAAGAGATATAGGTCAACTGCTAGGACTACCTATACCTTTGCGAAGCCAATGGCTGCTAACTATCTTAAAAATCAGCCCATGTACGTTTTCAGGAAAACAGAGCTAAAGCATAGCAAAACAGAATTGAATTTTAAGGAATGGCAAAAGGCTTTTACGGATGTTATGGGAATGGACGAGCTATACAAAGGATCCGGTGGTGGAGAGGGAAGGGGGTCCCTTCTTACATGTGGCGATGTAGAAGAAAATCCGGGGCCTATGGCTCCATTGTCACAGGAAGAAAGCACATTGATAGAGAGAGCCACTGCTACGATAAATAGCATTCCTATCTCAGAGGACTATTCTGTCGCTTCCGCCGCGTTATCTTCAGATGGTCGTATATTTACGGGAGTCAACGTATACCATTTTACTGGTGGACCCTGCGCAGAGTTAGTGGTCCTTGGTACTGCCGCAGCAGCGGCGGCGGGCAACCTTACCTGCATCGTAGCAATCGGAAATGAAAATAGAGGCATATTGTCACCGTGTGGGAGATGCCGTCAGGTTTTATTGGATTTGCACCCCGGCATTAAAGCCATAGTCAAGGACTCTGATGGGCAGCCTACAGCCGTGGGAATCAGAGAATTGCTTCCGTCAGGGTACGTGTGGGAGGGCTAATAAGGTACCACCTAACAACTTTATGTCGACTC

Donor sequences were constructed by cloning a synthetic DNA fragment encoding mNeon green and the downstream T2A and BSD sequences (IDT) into pDCIn (DiCre induction) plasmid that had been linearised by digestion with MluI and KpnI (Patel et al., 2019).

**Supplemental Movie 1:**

Both DMSO (Hoechst) and RAPA (DIC) treated parasites egress normally. Percentage of egress: DMSO: 36.5% (23/63), RAPA: 35.8% (33/92). Scale bars, 10 μm.

### Supplemental Table 1:

### Proteins identified from pull down experiments of PP7-HA:loxP and 3D7DiCre in P. falciparum schizonts.

### Supplemental Table 2:

### Phosphoproteome analyses of PP7-HA:loxP and 3D7DiCre in P. falciparum schizonts.

**References**

ANDREEVA, A. V. & KUTUZOV, M. A. 2009. PPEF/PP7 protein Ser/Thr phosphatases. *Cell Mol Life Sci,* 66**,** 3103-10.

BARTON, G. J., COHEN, P. T. & BARFORD, D. 1994. Conservation analysis and structure prediction of the protein serine/threonine phosphatases. Sequence similarity with diadenosine tetraphosphatase from Escherichia coli suggests homology to the protein phosphatases. *Eur J Biochem,* 220**,** 225-37.

BIRNBAUM, J., FLEMMING, S., REICHARD, N., SOARES, A. B., MESEN-RAMIREZ, P., JONSCHER, E., BERGMANN, B. & SPIELMANN, T. 2017. A genetic system to study Plasmodium falciparum protein function. *Nat Methods,* 14**,** 450-456.

KERK, D., MATTICE, J. F., VALDES-TRESANCO, M. E., NOSKOV, S. Y., NG, K. K. & MOORHEAD, G. B. 2021. The origin and radiation of the phosphoprotein phosphatase (PPP) enzymes of Eukaryotes. *Sci Rep,* 11**,** 13681.

PATEL, A., PERRIN, A. J., FLYNN, H. R., BISSON, C., WITHERS-MARTINEZ, C., TREECK, M., FLUECK, C., NICASTRO, G., MARTIN, S. R., RAMOS, A., GILBERGER, T. W., SNIJDERS, A. P., BLACKMAN, M. J. & BAKER, D. A. 2019. Cyclic AMP signalling controls key components of malaria parasite host cell invasion machinery. *PLoS Biol,* 17**,** e3000264.
